# A source-sink model explains the difference in the metabolic mechanism of mechanical damage to young and senescing leaves in *Catharanthus roseus*

**DOI:** 10.1186/s12870-021-02934-6

**Published:** 2021-03-26

**Authors:** Qi Chen, Xueyan Lu, Xiaorui Guo, Mingyuan Xu, Zhonghua Tang

**Affiliations:** 1 School of Life Sciences Nantong University, Nantong, 226010 P. R. China; 2 Northeast Forestry University, Harbin, 150040 P. R. China; 3 First Affiliated Hospital, Heilongjiang University of Chinese Medicine, Harbin, 150040 P. R. China

**Keywords:** Mechanical damage, Metabolomics, *Catharanthus roseus*, Source-sink model, Resource trade-off

## Abstract

**Background:**

Mechanical damage is an unavoidable threat to the growth and survival of plants. Although a wound to senescing (lower) leaves improves plant vitality, a wound to younger (upper) leaves often causes damage to or death of the whole plant. Source-sink models are often used to explain how plants respond to biotic or abiotic stresses. In this study, a source-sink model was used to explain the difference in the metabolic mechanism of mechanical damage to young and senescing leaves of *Catharanthus roseus*.

**Results:**

In our study, GC-MS and LC-QTOF-MS metabolomics techniques were used to explore the differences in source-sink allocation and metabolic regulation in different organs of *Catharanthus roseus* after mechanical damage to the upper/lower leaves (WUL/WLL). Compared with that of the control group, the energy supplies of the WUL and WLL groups were increased and delivered to the secondary metabolic pathway through the TCA cycle. The two treatment groups adopted different secondary metabolic response strategies. The WLL group increased the input to the defense response after damage by increasing the accumulation of phenolics. A source-sink model was applied to the defensive responses to local (damaged leaves) and systemic (whole plant) damage. In the WUL group, the number of sinks increased due to damage to young leaves, and the tolerance response was emphasized.

**Conclusion:**

The accumulation of primary and secondary metabolites was significantly different between the two mechanical damage treatments. *Catharanthus roseus* uses different trade-offs between tolerance (repair) and defense to respond to mechanical damage. Repairing damage and chemical defenses are thought to be more energetically expensive than growth development, confirming the trade-offs and allocation of resources seen in this source-sink model.

**Supplementary Information:**

The online version contains supplementary material available at 10.1186/s12870-021-02934-6.

## Background

Plants are subjected to various biotic and abiotic stresses in the natural environment during their growth and development [1]. Wounding is common mechanical damage to plants that occurs because of abiotic and biotic stress, which threatens plant growth and survival [2]. Mechanical damage disrupts the integrity of cells in plants. It causes cell membrane disruption, desiccation, lipid and protein oxidation, and protein aggregation [3]. Damaged tissues lead to plant nutrient loss and pathogen invasion, causing the disease to spread throughout the plant [2]. Plants have developed constitutive and induced defense mechanisms to respond to wounding and prevent infection properly [4]. Previous studies have shown that environmental stresses stimulate the accumulation of primary and secondary metabolites, which protect plants against pests, diseases, and stresses [5–8]. Specific metabolites are concentrated on the wound, promotes wound healing and prevents microbial infection. This is caused by the mechanical damage-induced activation and regulation of specific metabolic pathways [9]. The related metabolites help plants resist the wounding caused by herbivores, pathogens, or competitors through direct or indirect toxic effects [10]. Therefore, the changes in metabolites and metabolic pathways reflect the response of plants to mechanical damage.

Source-sink models have explained the relationship between plant demand changes and energy allocation, which involves complex metabolic and signaling networks [11–13]. Plants often reconfigure their nutritional resources and secondary metabolites in response to environmental stresses [10, 14]. Metabolite changes lead to a redistribution of internal plant resources between growth and defense [15]. Growth patterns are derived from the interaction between source processes (those that supply carbohydrates, the plant’s building blocks) and sink activity (the demand for carbohydrates) [16]. Wounded plants experience physiological changes when the resources required for defense exceed those required for growth and reproduction. These changes include activating dormant meristems, altering plant structure and growth, and regulating resource allocation between storage and reproduction [17, 18]. Carbohydrate partitioning is the process of carbon assimilation and distribution from source tissues, such as leaves, to sink tissues, such as stems, roots, and seeds [19]. Carbohydrate regulation genes influence sugar metabolism, adjust resource allocation for plant responses to stress and variations in signals from the environment [20, 21]. Gene expression changes the source and sink activities in plants to regulate their growth patterns based on the availability and acquisition of carbon resources [10]. Carbon levels in storage organs influence the net photosynthetic activity in source tissues, whereas sugar levels change photosynthesis-related enzyme expression in leaves. However, the mechanisms whereby sugars regulate source gene expression in plants remain relatively unexamined [22].

*Catharanthus roseus* (*C. roseus*), a member of the Apocynaceae, is an ornamental plant in botanical gardens [23]. This plant produces various secondary metabolites during its growth [24] and is a medicinal plant model for secondary metabolism studies [25]. Terpenoid indole alkaloids (TIAs) are the essential secondary metabolites of the species [26]. These metabolites have distinctive chemical structures, comprising indole and a terpenoid moiety, which helps plants protect against biological and environmental stress [27, 28]. Moreover, *C. roseus* contains vinblastine, vincristine, and other alkaloids, which can effectively inhibit tumors, making it the most widely used natural antitumor ingredient extracted from plants. The sulfate composition of the plant has been widely used in practical medicine [29].

In the present study, we researched the defensive strategy in *C. roseus* after mechanical damage to young (upper) and senescing (lower) leaves. GC-MS and LC-MS were used to detect and identify metabolites and provide insight into the metabolic pathway of mechanical damage regulation [30–33]. We hypothesized that the metabolite changes in *C. roseus* after leaf mechanical damage were related to the trade-off and source-sink relationship. For this purpose, we analyzed the metabolites and associated gene changes in different organs. The metabolomics strategy was used to analyze the changes in metabolic pathways. Our research can increase the understanding of the defense mechanisms of plants, thus providing a basis for research on different metabolic responses to mechanical damage.

## Results

### Responses of primary metabolites

**Fig. 1:**
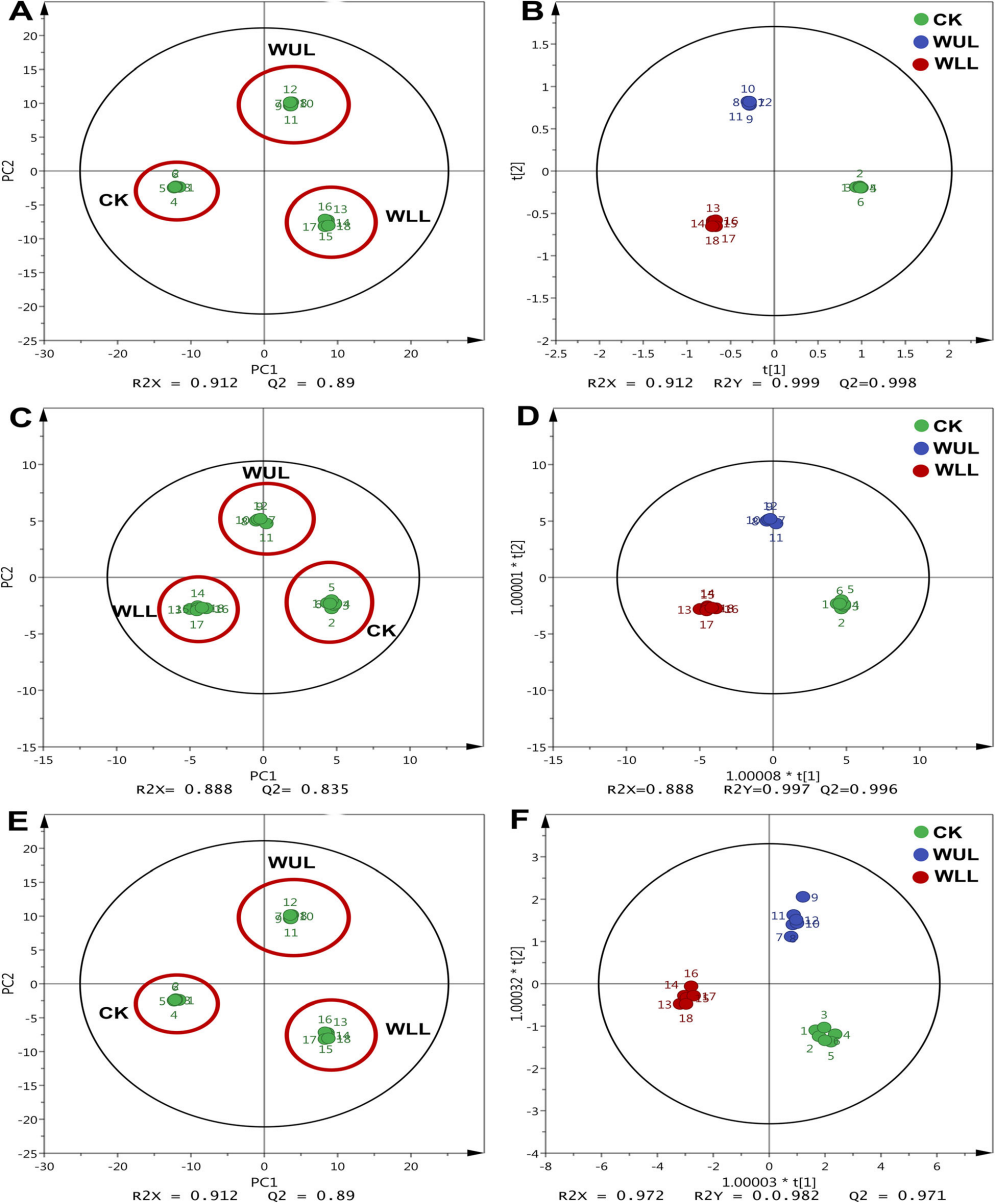
The PCA and PLS-DA score plot of primary metabolism, TIAS, and PCs of mechanical wounding: **a**: PCA score plot of primary metabolites; **b**. PLS-DA score plot of primary metabolites; **c**: PCA score plot of phenolic metabolites; **d**. PLS-DA score plot of phenolic metabolites; **e**: PCA score plot of alkaloid metabolites; **f**. PLS-DA score plot of alkaloid metabolites; CK: Control group, WUL: damaged upper leaf group, WLL: damaged lower leaf group

One hundred seventy-four metabolites were detected by using GC-MS. PCA models were used to analyze the differences in primary metabolites between treatment groups. The results showed that the three treatment groups had significant metabolic differences (Fig. 1. a). The OPLS-DA model was used to identify the different metabolites between the treatment groups (Fig. 1. b). Sixteen different metabolites were found depending on their VIP values (VIP > 1) and *P*-values (*P* < 0.05) (Table S1). Each metabolite is marked in the KEGG database to find the biological pathways involved. The significance criterion was *p* ≤ 0.05 as the screening criterion for differential metabolic pathways. Galactose metabolism and fatty acid biosynthetic pathways were marked by the significance criterion *p* ≤ 0.05 (Table 1), consisting of four sugars and two fatty acids (Table S2).

**Table 1:** Metabolic pathways enriched by significantly different metabolites (GC-MS)

KEGG	*P*-value	Enriched by significantly different metabolites	VIP	Change
Galactose metabolism	1.91E-04**	D-Fructose	1.44	CK > WUL > WLL
		Galactitol	1.03	WUL > CK > WLL
		Glycerol	1.73	CK > WUL > WLL
Fatty acid biosynthesis	8.71E-03**	Tetradecanoic acid	1.27	CK > WLL > WUL
		Octadecanoic acid	1.76	CK > WLL > WUL

*P*-value, Significantly *P < 0.05, Extremely significantly **P < 0.01; *VIP* variable importance in the projection, *CK* Control group, *WUL* damaged upper leaf group, *WLL* damaged lower leaf group

**Fig. 2:**
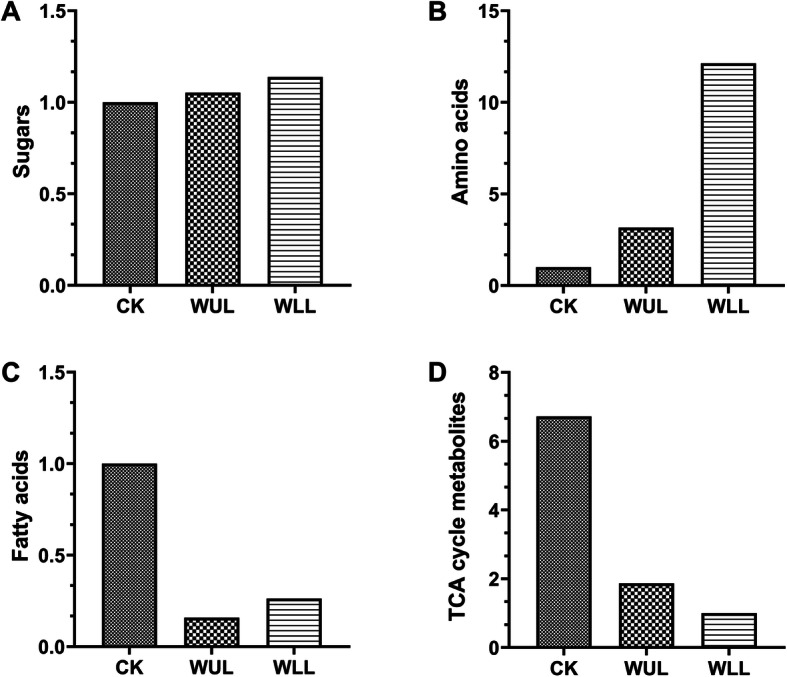
The relative content Q-values for major primary metabolites were analyzed using GC-MS: **a**. Sugars, **b**. Amino acids, **c**. fatty acids, **d**. TCA cycle metabolites; Control group, CK; Damaged upper leaf group, WUL; Damaged lower leaf, WLL

The Q value showed the overall accumulation of primary metabolites. Mechanical damage increased the sugars, and amino acids accumulated in *C. roseus*. Sugars in the WUL and WLL were 5.25 and 13.74% higher than those in the CK (Fig. 2. a). Amino acids in the WUL and WLL were 215.30 and 1213.00% higher than those in the CK (Fig. 2. b). In contrast, mechanical damage reduced fatty acids, and TCA cycle metabolites accumulated. Fatty acids in the WUL and WLL were 84.21 and 73.68% lower than those in the CK (Fig. 2. c). The TCA cycle metabolites in the WUL and WLL were 572.00 and 87.00% lower than those in the CK (Fig. 2. d).

### Responses of phenolics

**Fig. 3:**
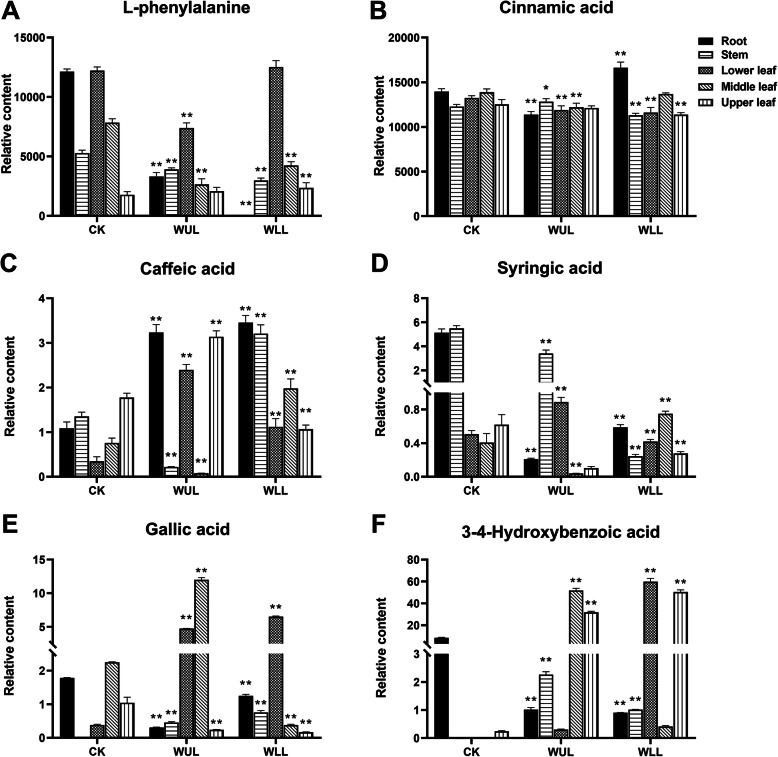
Significantly different changes in L-phenylalanine, C6C1, and C6C3-type PCs: CK: Control group, WUL: damaged upper leaf group, WLL: damaged lower leaf; group; *n* = 6, “**” means that there is an extremely significant difference between the treatment group and the control group (*p* < 0.01), “*” means that there is a significant difference between the treatment group and the control group (*p* < 0.05)

**Fig. 4:**
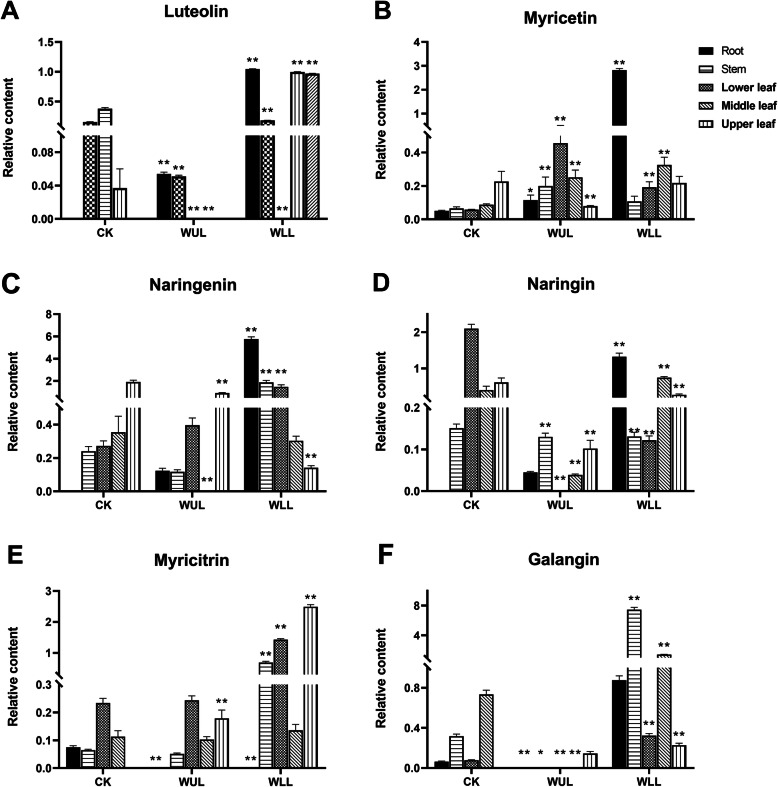
Significantly different changes in C6C3C6-type PCs: CK: Control group, WUL: damaged upper leaf group, WLL: damaged lower leaf; group; *n* = 6, “**” means that there is an extremely significant difference between the treatment group and the control group (*p* < 0.01), “*” means that there is a significant difference between the treatment group and the control group (*p* < 0.05)

The PCA model showed that phenolic accumulation was different in the treatment groups (Fig. 1. c). The OPLS-DA model obtained 11 significantly different PCs (VIP > 1, *P* < 0.05) (Fig. 2. d, Fig. 3, Fig. 4). Phenylalanine in the WLL and WUL was lower than that in the CK in roots, stems, and median leaves. It was higher than in the CK in the upper leaves (Fig. 3. a). Cinnamic acid showed organ variation. In WLL, the aboveground organ content was lower than that in CK, while the roots showed the opposite trend. In WUL, cinnamic acid was lower than CK in roots and leaves, in stems was higher than CK (Fig. 3. b). Caffeic acid in the treatment groups was higher than CK except for the lower leaves. The stems and lower leaves were higher in WUL than WLL, WLL was higher than WUL in the middle leaves (Fig. 3. c). Syringic acid in WUL was lower than CK except for the lower leaves, and the median leaf in WLL was higher than CK (Fig. 3. d). In WUL, gallic acid was higher than CK except for roots and upper leaves. In WLL, its content was higher than CK in the root, stem, and lower leaves and lower than CK in the middle - and upper leaves (Fig. 3. e). 3–4-Hydroxybenzoic acid in the WUL was lower than that in the CK in underground organs. Except for the median leaf, the aboveground organs were higher than those in CK (Fig. 3. f).

In most organs, the response of luteolin, naringenin, naringin, and galangal in WLL was more robust (Fig. 4. a. c. d. f). Luteolin in WLL was lower than CK in all organs except the lower leaves. The content in WUL was lower than that in CK only in the upper leaves (Fig. 4. a). Myricetin in the roots and median leaves was higher in WLL than in CK. Except for the upper leaves, its content was higher than CK in WUL (Fig. 4. b). In WLL, the naringenin content was lower than that in CK in the middle and upper leaves. In WUL, its content was lower than that in CK in the middle and upper leaves (Fig. 4. c). In the WLL, the content of naringin in organs except the stem was higher than that in the CK. However, in WUL, its content was lower than that in CK in the stem and median leaves (Fig. 4. d). In the WLL, the aboveground organ myricitrin content was higher than that in the CK. In the WUL, its content was higher only in the upper leaves. In the root, myricitrin content was lower than CK in WLL and WUL (Fig. 4. e). The galangal content in WLL was higher than that in CK in all organs. In WLL, its content was lower than that in CK, except for the upper leaves (Fig. 4. f).

### The responses of TIAs

**Fig. 5:**
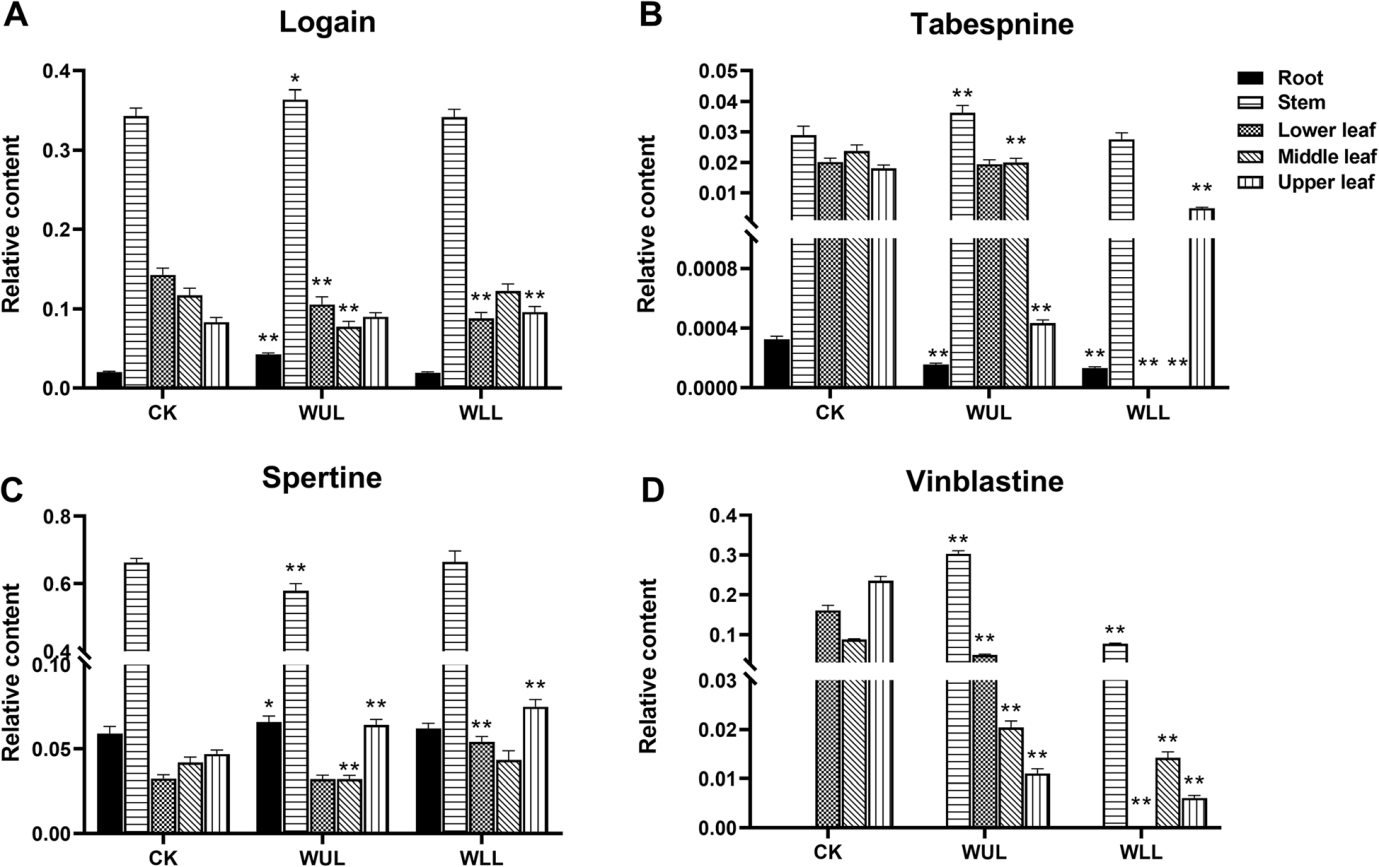
Significantly different changes in TIAs: CK: Control group, WUL: damaged upper leaf group, WLL: damaged lower leaf group; *n* = 6, “**” means that there is an extremely significant difference between the treatment group and the control group (*p* < 0.01), “*” means that there is a significant difference between the treatment group and the control group (*p* < 0.05)

The PCA model results showed that the TIAs were different in the treatment groups (Fig. 1. e). The OLPS-DA model showed four differential TIAs between the treatment groups (Fig. 1. f, VIP > 1, *P* < 0.05, Fig. 5). In WLL, the relative content of loganin was lower than that in CK in the lower leaves and higher than that in CK in the upper leaves. In WUL, its relative content was lower than CK in the lower and median leaves and higher than CK in roots and stems (Fig. 5. a). In WLL, the relative content of tabersonine was lower than that in CK in other organs except for the stem. In WUL, it was lower than CK in the root, median, and upper leaves (Fig. 5. b). In WLL, the relative content of spertine was higher in the lower and upper leaves than in CK. In WUL, it was higher in roots and upper leaves than in CK and lower in stems and median leaves (Fig. 5. c). In WLL, the relative content of vinblastine was higher in the stem than in CK and lower in the leaves. In WUL, it was lower than the CK in above-ground organs (Fig. 5. d).

### Influx direction of primary metabolic production

**Fig. 6:**
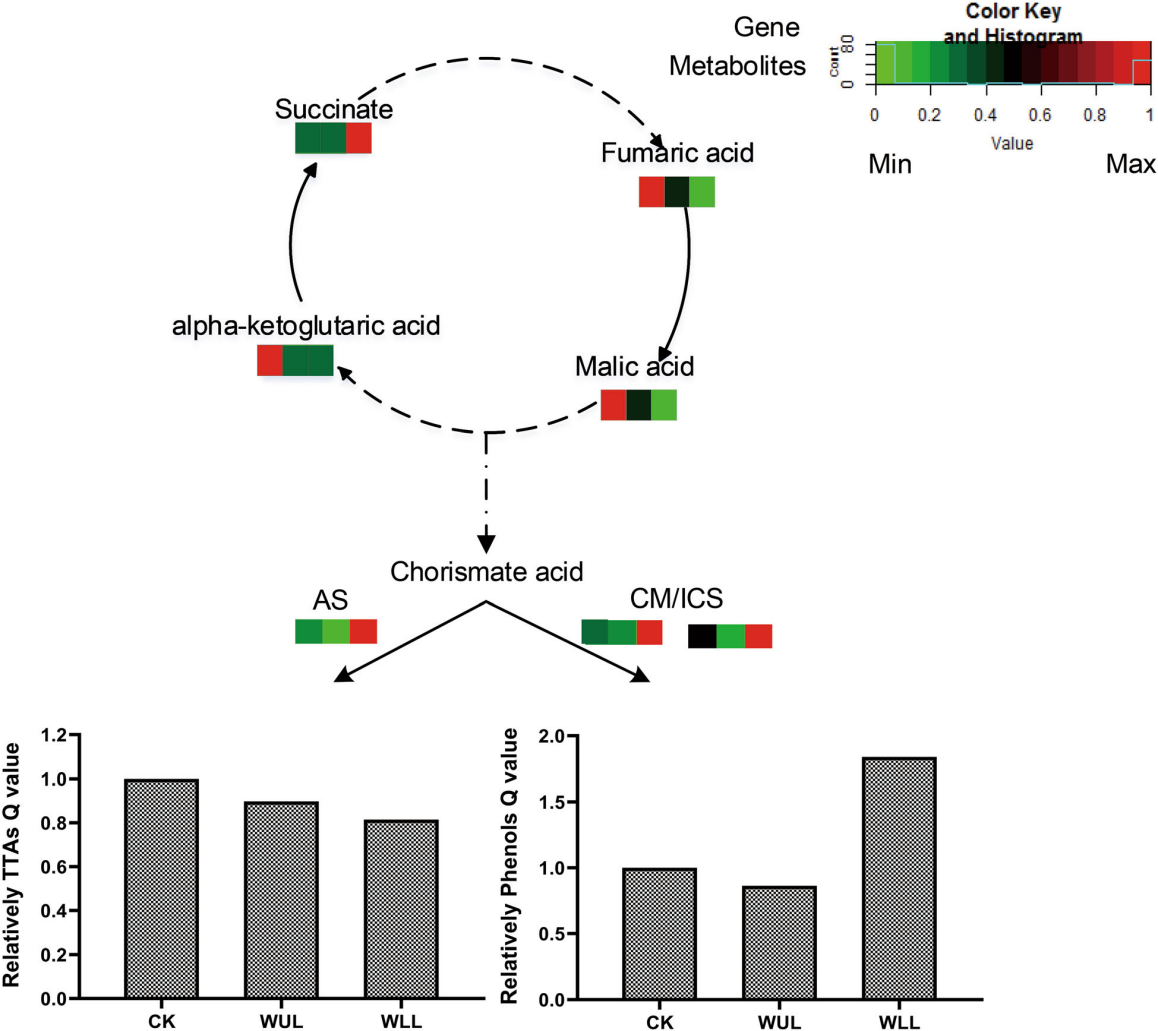
Metabolic allocation map. The grid shows the CK, WUL and WLL groups from left to right; the content from low to high is indicated by the color scale from blue to red, respectively; a solid line represents a one-step reaction, and a dotted line represents a multistep reaction; the TIA Q values and phenol Q values are represented in a histogram; CK: control group, WUL: damaged upper leaf group, WLL: damaged lower leaf group

**Fig. 7:**
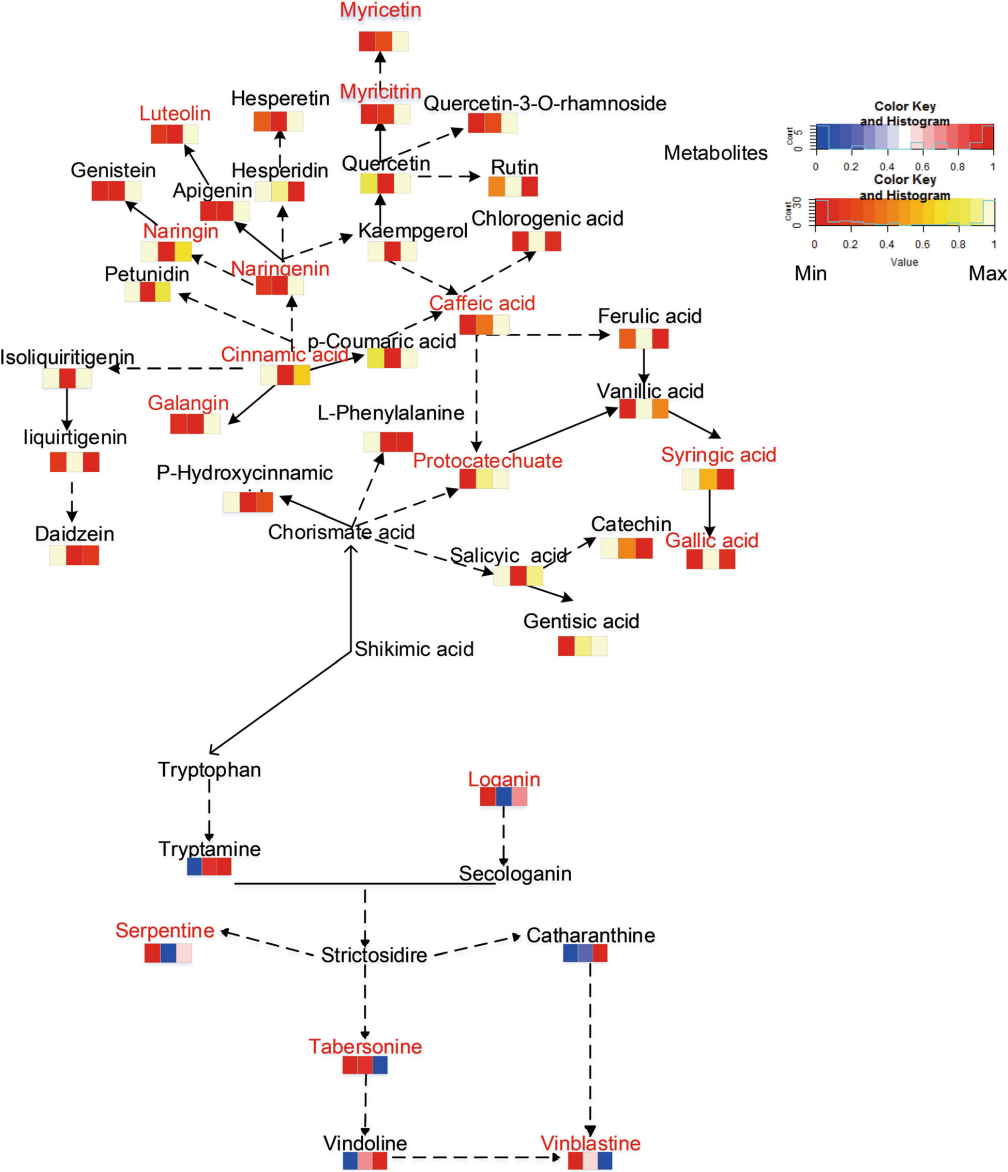
Diagram of the network of secondary metabolites. The grid shows the CK, WUL and WLL groups from left to right, respectively; the content of PCs from low to high is indicated by the color scale from red to white, respectively; the content of TIAs from low to high is indicated by the color scale from blue to red, respectively; a solid line represents a one-step reaction, and a dotted line represents a multistep reaction; CK: control group, WUL: damaged upper leaf group, WLL: damaged lower leaf group

WLL consumed more energy in the TCA cycle than CK. More resources flowed into secondary metabolism through the TCA cycle. Compared with WUL, genes that were more highly expressed in WLL were anthranilate synthase (AS), chorismate mutase (CM), and isochorismate synthase (ICS) (Fig. 6). Although the expression of genes related to TIA synthesis increased, the Q value of TIAs in the mechanical damage treatment group decreased (Fig. 7). This result suggests that the decrease in TIA accumulation may be due to the inhibition of synthesis. We also found a correlation between TIA gene expression and PC accumulation. When the TIA genes were expressed at high levels, the PCs accumulated more in the WLL. Low TIA gene expression corresponded to lower PC accumulation in the WUL (Fig. 6).

### The response of a metabolic network map

In terms of primary metabolism, mechanical damage promoted carbohydrate and amino acid metabolism and inhibited TCA cycle metabolism (Fig. S3). The metabolic changes in the WLL group were more pronounced than those in the WUL group (Fig. S3). Regarding secondary metabolism, mechanical damage affects PC metabolism. WLL promoted the branching pathways of galangal, naringenin, caffeic acid, and myricetin (Fig. 7). The TIA synthesis pathway was also affected. The tryptamine in the synthetic upstream region of TIA was higher in the treatment group than in the CK group. However, loganin was lower than CK. The synthesis of downstream metabolites in TIA synthesis pathways has also been affected. Vincristine was lower in the treatment group than CK. In WLL, its precursor metabolite tabersonine was also reduced. However, the contents of catharanthine and vindoline were higher than those in CK. In the WUL, the content of the three precursor metabolites of vinblastine was higher than that in the CK.

## Discussion

Metabolism is a general term for all aspects of the physiological, biological, and functional regulation of compound synthesis [34]. Primary metabolism is the plant regulation and distribution of nutrients and energy and provides the basis and raw material for secondary metabolism to provide resistance to environmental stress [35]. Nontargeted analysis of the WUL and WLL groups showed that many primary metabolites were synthesized after mechanical damage, and most of them were sugars (Fig. 2). In this process, carbohydrates are mainly used as substrates for energy production and secondary metabolic materials to increase plant resistance, reflecting that plants have a high demand for energy after mechanical damage stress. KEGG pathway enrichment analysis identified carbohydrate metabolism, which is closely related to the response to mechanical stress of the plant. Sugar metabolism, glycolysis, and the TCA cycle are all carbon metabolism pathways directly related to supplying available energy and carbon skeletons during the growth and development of plant life activities [36]. The TCA cycle is the final oxidative pathway for carbohydrates, fats, and amino acids, which is an important metabolic pathway for the energy supply to plants [37]. In this study, TCA cycle metabolites participated in the central metabolic reconstruction of related metabolites in the treatment group after mechanical damage stress. Succinate accumulated more in the WLL group, malic acid and fumaric acid accumulated more in the WUL group, entering into different carbohydrate metabolism (Fig. 7).

Amino acids are essential metabolites in plant primary metabolism and play an important role in plant physiological processes: acting as osmolytes, modulating stomatal opening, and serving as precursors for the synthesis of defense-related metabolites and signaling metabolites [38]. An enormous variety of secondary metabolites are derived from their chemical structures, many of which have important defense mechanisms [39]. The Q value of amino acids among treatment groups showed that the WLL had the strongest response to mechanical damage, which resulted from the common response of different tissues in the plant after the senescing (lower) leaves were damaged (Fig. 2. b). In addition, fatty acids play an important role in plant resistance [40]. They were significantly reduced in the WLL group (Fig. 2. c), indicating that the plant actively mobilizes primary metabolism to prepare for damage to the senescing leaves.

Plant growth stage, environmental stress, nutrition, and plant genetics influence the production of secondary metabolites [41]. PCs are widespread in plants and thought to act as physical barriers and antibacterial substances [42]. They are an interconnected network metabolism and respond to environmental stress through regulation and cooperation [43]. Protocatechuic acid is the precursor of tannin with a special defensive effect in plants [44]. The downstream metabolite of quercetin-3-o-rhamnoside can effectively promote wound closure [45]. Hesperidin is effective against the invasion of plant pathogens [46]. We found that more PCs responded positively to mechanical damage in the WLL group (Fig. 7). Protocatechuic acid and quercetin-3-o-rhamnoside only responded in the WUL group. It should be noted that only hesperidin responded locally in the WLL group, while other PCs involved in the response of each treatment group responded systematically. Finally, we can better play the mechanical damage response role through local or system response trade-offs. Therefore, PCs in different tissues have taken different strategies to respond to mechanical damage stress.

TIAs and PCs were the major secondary metabolites in plants. PCs are synthesized via the phenylpropanoid pathway [47]. Hence, they compete with TIA biosynthesis for the common precursor chorismate [48]. TIAs and PCs in the trade-off allocation to cooperate or compete with plants respond to biotic and abiotic stresses [49]. After preliminary energy and raw material preparation, different treatments first responded to PCs. Then, TIAs responded (Fig. 6). There are time series differences between them. The migration of metabolites in plants has nothing to do with the biomass of tissues and organs and is considered source-sink relationship regulation [50]. Source-sink models have been gradually applied to control plant resources under stress [13, 51]. In this study, mechanical damage destroys the source-sink balance. Therefore, the damaged plants must establish a new source-sink balance to reduce the consumption of other parts of the resource allocation.

Compared with WLL, WUL had more synthetic disorders and lower secondary metabolic input. Because young (upper) leaves are the key tissue parts of plant growth and development, mechanical damage to young leaves will produce more negative effects on the plant and consume more resources and energy allocation. As the redundant tissue part of plants aging, damage to senescing leaves often brings more sufficient resource allocation and beneficial stimulation to plants. The results showed that WUL had more negative effects on *C. roseus*, w*hich* readjusted the resource allocation through the source-sink model after mechanical damage, effectively changing source and sink activities (Fig. 7, Fig. S3). Plants can adjust their growth patterns according to the availability of carbon resources [52]. That is, after damage, the young leaves changed from a strong source to a strong sink, which required more resources.

The factors leading to the sink strength increase in mechanical damage stress include the repair process (tolerance) and secondary metabolism regulation (resistance). Different treatments lead to different responses and coping strategies. After plants suffer from damage, they first consume carbon-based primary metabolites and then choose tolerance or resistance through trade-offs [53]. Our study found that the WLL group was mainly resistant, while the WUL group was mainly tolerant. After mechanical damage, plants still selectively carry out secondary metabolism under resource limitation, inducing related defense compounds, reducing resource utilization in the repository, and showing the importance of secondary metabolism in response to mechanical damage [54, 55]. Therefore, damaged *C. roseus* must be effectively balanced and allocated under limited resources. The potential benefits of this division of labor are to minimize the metabolic cost caused by injury and ensure the implementation of the most effective defense strategy.

The results showed that mechanical damage changed primary and secondary metabolite accumulation in *C. roseus*. Metabolites were transferred from source to sink. Changes in metabolites changed the balance of the strength between the source (undamaged organs) and sink (damaged organs). Therefore, the WLL group had more abundant resource allocation and defense metabolite accumulation. They will play a critical defensive role in the damaged area. The damaged plants use their resources to improve the maximum damage adaptability and put in the proportion of resources. This leads to a trade-off between physiological and metabolic investment and ultimately chooses tolerant or resistant to respond to stress. However, there will be a configuration effect of the plant trade-off allocation. An increase in one kind of metabolite will lead to a decrease in other metabolites. In our study, the PC response was the main response in the treatment group. There was a timing difference for the TIA responses. Therefore, the WLL group, with more induced resources, might have increased allocation to self-repair.

## Conclusion

In conclusion, the changes in the primary and secondary metabolites in *C. roseus* under mechanical damage stress resulted from metabolic pathway alterations, which varied depending on the growth stage of the damaged leaves. The results indicate that mechanical damage will cause primary metabolic resources and the redistribution of secondary metabolites in *C. roseus*. A source-sink model was proposed to explain this mechanism of resource reallocation. The results provided useful information for studying plant responses to mechanical damage in different ways and exploring new plant models of responses to adverse ecological conditions and stress. Although we have connected the flow of resources for metabolic in series under mechanical damage, the mechanisms of repair damage by metabolic responses are not fully explained. We will focus on cellular damage in future studies, and attempt to connect the leaf damage/tolerance/repair with metabolic/molecular responses. Thus, the system response mode from the induction of apparent damage to internal metabolic regulation to repair external damage will be revealed.

## Methods

### Plant material and treatment

The *C. roseus* seeds were purchased from Guangdong Shicheng Farm Co., Ltd. (Guangzhou, Guangdong, China). The purchased seed identity was verified by Prof. Zhonghua Tang (Key Laboratory of Plant Ecology, Northeast Forestry University). Seed specimens were deposited at the Key Laboratory of Plant Ecology, Northeast Forestry University. The seeds were grown in a growth chamber with temperature conditions at a 27–28 °C/23–25 °C day/night temperature cycle, 75% relative humidity, and a 12-h light/12-h dark photoperiod cycle. After germination, the seedlings were irrigated with 1/2 strength Hoagland solution (pH 5.9–6.0). After growing 7–8 leaves (3 months later), each mature plant was separated into five different physiological areas or sections: roots, stems, lower leaves, middle leaves, and upper leaves.

There are three treatment groups in this study. The control group (CK) was without mechanical damage treatment. The upper (young) leaves and lower (senescing) leaves were treated with mechanical damage by sterilized scissors, WUL and WLL, respectively. As shown in Fig. S1, the distribution diagram shows the treatment position in *C. roseus*. Six biological replicates per treatment group were included for statistical analyses. The relationship between metabolite response and time was examined to find the optimum processing time. The samples were collected at 0 h, 0.5 h, 1 h, 3 h, and 5 h. The results showed that the most intense responses among the treatment groups were seen at 1 h (Fig. S2); therefore, all sampling times in the study were performed at 1 h.

### GC-MS analysis

Sixty milligrams of plant tissue were weighed and mixed with 360 μL of cold methanol and 40 μL of internal standards. The mixture was then homogenized using a tissue layer system and sonicated for 30 min. Afterward, 200 μL of chloroform and 400 μL of water were added. After an adequate response, the sample was centrifuged at 10,000 g for 10 min at 4 °C. Another 200 μL of chloroform and 400 μL of water were added. Samples were allowed to dry and then methoxyaminated and silylated [8].

After derivatization by the steps described above, samples were analyzed on the GC-MS system. A nonpolar DB-5 capillary column was used for separation. The carrier gas was high purity helium with a flow rate of 1.0 mL/min. The temperature program was 50–125 °C for 8 min, raised at 125–170 °C for 15 min, raised at 170–210 °C for 4 min, raised at 210–270 °C for 10 min, raised at 270–305 °C for 5 min, and maintained at 305 °C for 5 min. The inlet temperature was 260 °C, the EI source temperature was 230 °C, and the EI source voltage was − 70 V. Mass spectra were collected by scanning from 50 to 600 *m/z*, acquisition started after a 5 min delay, and the acquisition speed was 20 spectra/s.

### LC-QTOF-MS analysis

Fresh plant tissue was first freeze-dried in a refrigerator at − 180 °C and then pulverized. One gram of the pulverized sample was weighed and dissolved in 20 ml of methanol and subsequently low-frequency ultrasonicated for 40 min. The simple solution was centrifuged for 10 min at 8000 rpm. TIAS detection was based on a Waters ACQUITY UPLC system (Waters, JAPAN) and a Qtrap 5500 ion trap mass spectrometer (ABS, SCIEX, USA) equipped with an ACQUITY UPLC BEH C18 column. The column temperature was 30 °C. The flow rate was 0.25 ml/min. The UPLC mobile phase consisted of water (0.1% formic acid) (A) and acetonitrile (B). MS was an electrospray ion source with positive and multiple reaction monitoring scanning modes. The MS conditions were as follows: ion spray voltage, 5500 V; atomization temperature, 500 °C; atomizing air pressure, 25 psi; and air curtain pressure, 20 psi.

Targeted analysis of phenolic metabolites (PC_S_) was performed using a Waters ACQUITY UPLC system (Waters, Japan) coupled to a quadrupole time-of-flight (QTOF) mass spectrometer (XEVO G2 QTOF, Waters). Chromatographic columns: ACQUITY UPLC-BEH C18 column (1.7 mm, 2.1 mm, × 50 mm). The mobile phase system consisted of 0.05% formic acid-water (A) and 0.05% formic acid-acetonitrile (B). Multiple reaction monitoring transitions in positive mode were performed at 120–1200 m/z; the internal standard was Leu-enkephalin [26].

### The quantitative real-time PCR analysis

The conditions of quantitative real-time PCR (qRT-PCR) analysis were based on our previous research [56]. All plant samples were collected in six biological replicates and immediately frozen in liquid nitrogen. Total RNA was derived from the frozen samples using TRIzol Reagent (Invitrogen, USA). DNA contamination in the total RNA was removed using DNase I (New England Biolabs). The purity of DNA and RNA was detected by 1% agarose gel electrophoresis. The total RNA concentration was then detected using a NanoDrop spectrophotometer (Thermo Fisher Scientific, Inc.). Total RNA (2 g) was converted into cDNA using ReverTra Ace qPCR RT Master Mix (Toyobo, Shanghai, China) with oligo (dT) as a primer. qRT-PCR analysis using cDNA as a template and gene-specific primers were performed using SYBR Premix Ex Taq with initial denaturation. The PCR process included 95 °C for 30 s, followed by 35 cycles at 94 °C for 30 s, 56 °C for 30 s, and 72 °C for 30 s. This process was repeated at least three consecutive times for each sample to ensure reproducibility. The gene-specific primers used are listed in Table 2 (https://www.ncbi.nlm.nih.gov/genome/?term=txid4058, *C. roseus* genome). Ribosomal protein subunit 9 (Rsp9) was used as an internal control to evaluate all *C. roseus* plants [57, 58].

**Table 2:** Primers for qRT-PCR analyses of *C. roseus*-related genes [57]

Gene	Primer sequences
ICS	ATTGCAGACGATCGTTTAACTC TTCCTCGGTCAAACATTTCG
PAL	GGCCACCAAGATGATCGA CAATGGCCAATCTTGCATTG
CM	CGATTTGTTGAAATTGCAGACG ATTGCAGACGATCGTTTAACTC
C4H	GCCGATTCTCTGTATCACTATC ATGATTAAAATGATCTTGGCTTT
AS	GCGAACATTTGCAGATCCAT GGCCGATTTGTTATTGTTCC

*ICS* Ischorismate synthase, *PAL* Phenylalanine ammonia-lyase, *CM* chorismate mutase, *C4H* cinnamate 4-hydroxylase, *AS* anthranilate synthase

### Statistical analysis

The GC-MS raw data files were converted into CDF format (NetCDF) netCDF (*.cdf) format with Agilent GC/MS 5975 data analysis software (version 14.1, Agilent Technologies, Santa Clara, CA, USA) and were subsequently processed by XCMS (www.bioconductor.org) [59]. The normalized data were imported into SIMCA-P software (version 13.0, http://www.umetrics.com/simca). An unsupervised principal component analysis (PCA) analysis was applied to visualize the global metabolic profiles among groups. Next, supervised orthogonal partial least squares discriminant analysis (OPLS-DA) was used to identify differential metabolites between treatment groups. By OPLS-DA analysis, the differential metabolites responsible for discriminating between the two treatment groups were identified with variable importance plot values of greater than 1.0 and *P* values of less than 0.05. Differential metabolites were annotated using the KEGG database (http://www.kegg.jp/kegg/pathway.html) and MBRole 2.0 (http://csbg.cnb.csic.es/mbrole2/) [60]. The score of principal component “Q” (Q) was calculated using Statistical software SPSS version 21.0 software (Chicago, IL, USA) and was used for the score of principal component “Q value” (Q) statistical analysis. Q is used as an indicator of the comprehensiveness of the analysis and scientific evaluation of objective phenomena. Heat maps, histograms, and pathway maps were generated using GraphPad Prism version 6.0 (GraphPad Software Inc., La Jolla, CA, USA) or R version 3.5 (R Foundation for Statistical Computing, Vienna, Austria; https://www.R-project.org/).

## Supplementary Information

**Additional file 1: Figure S1.** The Pattern diagram of *Catharanthus roseus. ***Figure S2.** Effects of different treatment time on primary metabolites: Treatment time: 0 h, 0.5 h, 1 h, 3 h, and 5 h; Q value was combined with the treatment groups of CK, WUL, and WLL. **Figure S3.** The changes in the network of primary metabolism. The grid was CK, WUL and WLL group from left to right, respectively; the content of primary metabolites from low to high indicated by the color of green to red, respectively; CK: Control group, WUL: damaged upper leaf group, WLL: damaged lower leaf group. **Table S1**. Differential metabolites among treatment groups (GC-MS). **Table S2.** Differential metabolites (sugars and fatty acids) among treatment groups (GC-MS).

## Data Availability

The datasets used and/or analysed during the current study are available from the corresponding author on reasonable request.

## Abbreviations

*C. roseus*: *C. roseus*
PCs: Phenolics
TIAs: Terpenoid indole alkaloids
PCA: Principal component analysis
OPLS-DA: Discriminant analysis by orthogonal partial least square
CK: Control group
WUL and WLL: The upper leaves and lower leaves were treated with mechanical damage
