# Supplementary material for: A source-sink model explains the difference in the metabolic mechanism of mechanical damage to young and senescing leaves in Catharanthus roseus
Source: BMC Plant Biol. 2021 Mar 26;21:154. doi: 10.1186/s12870-021-02934-6 (PMC7995597; doi:10.1186/s12870-021-02934-6)
Supplement: Supplementary file 1 — Additional file 1: Figure S1. The Pattern diagram of Catharanthus roseus. Figure S2. Effects of different treatment time on primary metabolites: Treatment time: 0 h, 0.5 h, 1 h, 3 h, and 5 h; Q value was combined with the treatment groups of CK, WUL, and WLL. Figure S3. The changes in the network of primary metabolism. The grid was CK, WUL and WLL group from left to right, respectively; the content of primary metabolites from low to high indicated by the color of green to red, respectively; CK: Control group, WUL: damaged upper leaf group, WLL: damaged lower leaf group. Table S1. Differential metabolites among treatment groups (GC-MS). Table S2. Differential metabolites (sugars and fatty acids) among treatment groups (GC-MS). [file 12870_2021_2934_MOESM1_ESM.docx]

**Supplementary**


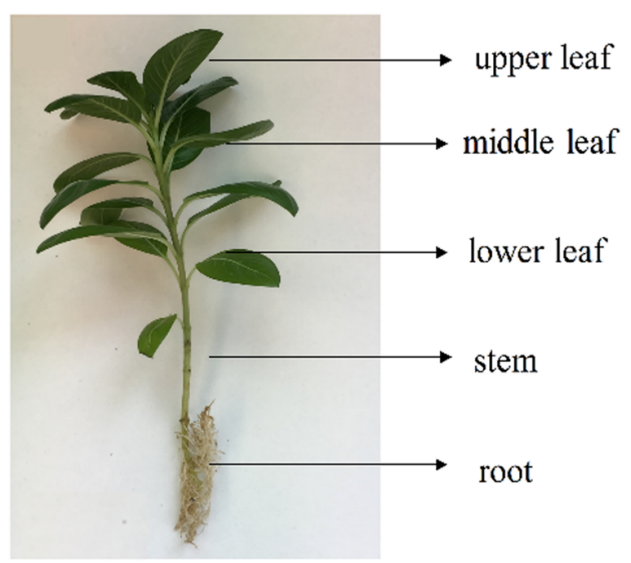


**Figure S1 The Pattern diagram of *Catharanthus roseus***


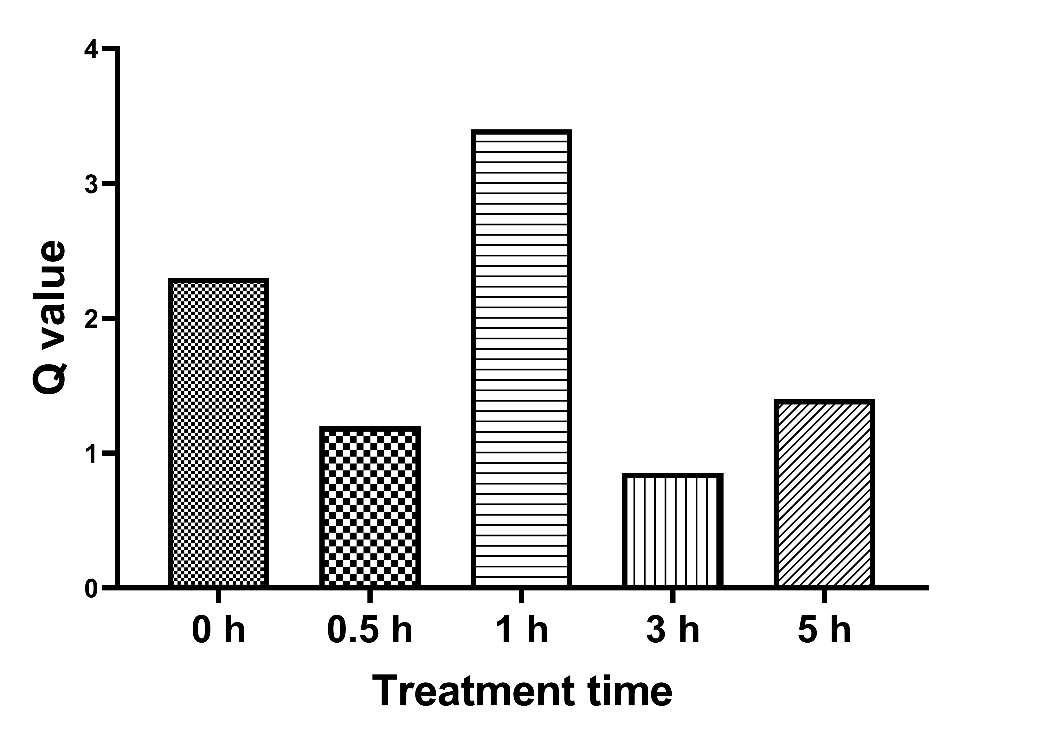


**Figure S2 Effects of different treatment time on primary metabolites::**

**Treatment time: 0 h, 0.5 h, 1 h, 3 h, and 5 h; Qvalue was combined with the treatment groups of CK , WUL, and WLL.**


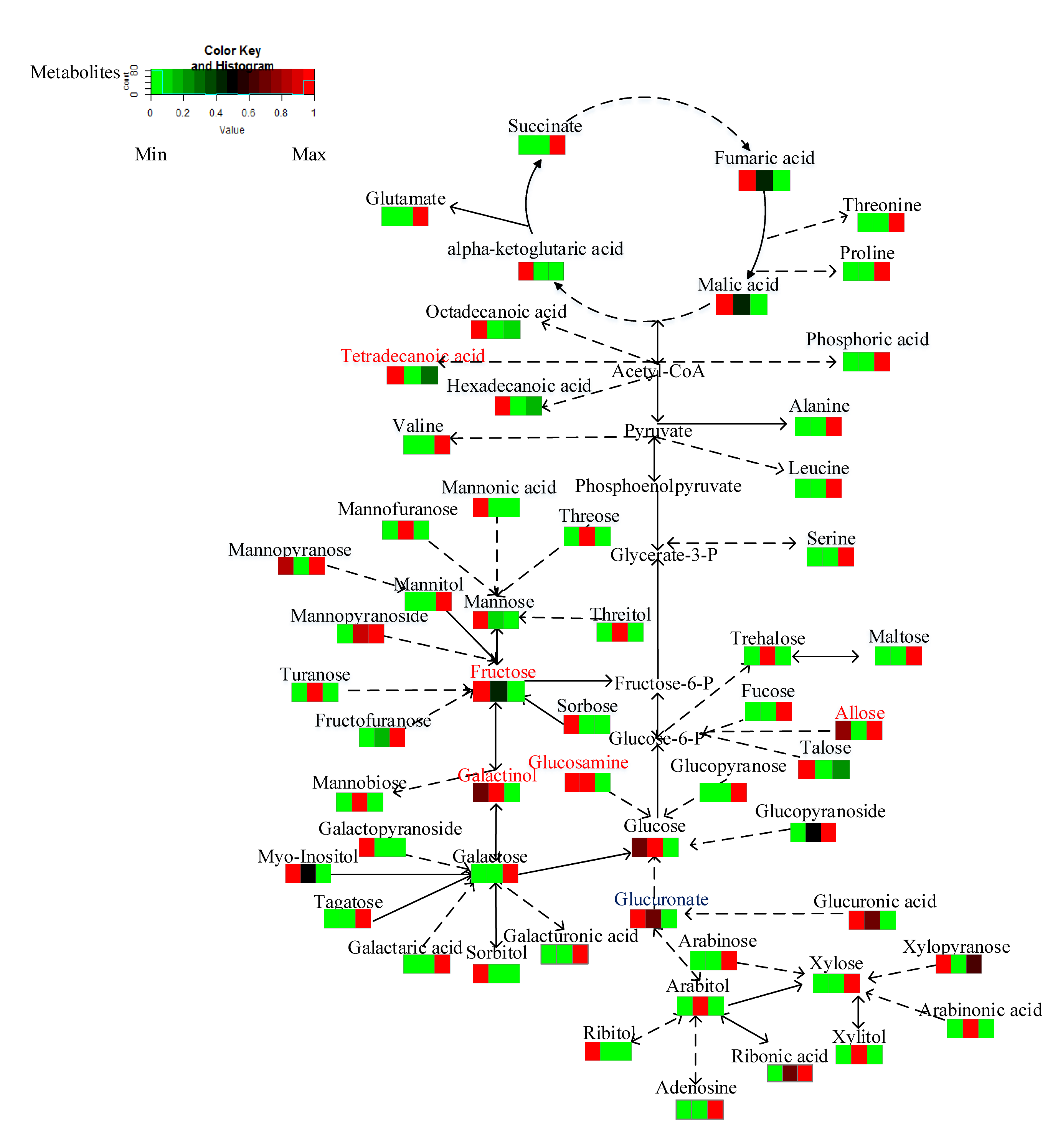


**Figure S3 The changes in the network of primary metabolism**

**The grid was CK, WUL and WLL group from left to right, respectively; the content of primary metabolites from low to high indicated by the color of green to red, respectively; CK: Control group, WUL: damaged upper leaf group, WLL:** **damaged lower leaf group.**

**Table S1. Differential metabolites among treatment groups (GC-MS)**

| Metabolite | Vip | *P*-value |
| --- | --- | --- |
| Carbamate | 2.51 | ** |
| Propanetricarboxylic acid | 1.77 | * |
| Octadecanoic acid | 2.81 | ** |
| Glycerol | 1.39 | * |
| Phenol | 1.34 | * |
| Benzoic acid | 1.21 | * |
| Fructose | 1.19 | ** |
| Tetradecanoic acid | 1.12 | * |
| Dimethylbenzyl isothiocyanate | 1.02 | * |
| Propanoic acid, | 1.02 | * |
| Butanoic acid | 1.21 | * |
| Phthalate | 1.05 | * |
| Glucosamine | 1.38 | * |
| Decanol | 1.13 | * |
| D-Allose | 1.54 | * |
| Galactitol | 1.78 | ** |

VIP, variable importance in the projection; Significantly: **P*<0.05, Extremely significantly: ***P*<0.01,

**TableS2. Differential metabolites (sugars and fatty acids) among treatment groups (GC-MS)**

|  | Metabolite | Vip | *P*-value |
| --- | --- | --- | --- |
| Sugars | Fructose | 1.19 | ** |
|  | Glucosamine | 1.38 | * |
|  | D-Allose | 1.54 | * |
|  | Galactitol | 1.78 | ** |
| Fatty Acids | Tetradecanoic acid | 1.12 | * |
|  | Octadecanoic acid | 2.81 | ** |

VIP, variable importance in the projection; Significantly: **P*<0.05, Extremely significantly: ***P*<0.01.
